# The Advancements of Marine Natural Products in the Treatment of Alzheimer’s Disease: A Study Based on Cell and Animal Experiments

**DOI:** 10.3390/md23030091

**Published:** 2025-02-20

**Authors:** Chunbo Jia, Jiaxin Chai, Shenyun Zhang, Yining Sun, Liheng He, Zhipei Sang, Dapeng Chen, Xu Zheng

**Affiliations:** 1 College of Basic Medical Sciences, Dalian Medical University, Dalian 116044, China; 2 Department of Comparative Medicine, Dalian Medical University, Dalian 116044, China; 3 Key Laboratory of Tropical Biological Resources of Ministry of Education, School of Pharmaceutical Sciences, Hainan University, Haikou 570228, China

**Keywords:** Alzheimer’s disease, marine natural products, Aβ aggregation, tau phosphorylation

## Abstract

As life expectancy rises and the aging population grows, Alzheimer’s disease (AD) has become a significant global health concern. AD is a complex neurodegenerative disorder with an unclear etiology. Current hypotheses primarily focus on β-amyloid (Aβ) aggregation, tau protein hyperphosphorylation, and neuroinflammation as key pathological processes. Given the limited efficacy of existing therapeutic strategies, there is an urgent need to explore novel treatment options. Marine natural products have garnered significant attention due to their unique chemical structures and diverse bioactivities, demonstrating potential for multi-target interventions in AD. This review systematically summarizes the roles of marine-derived compounds, including polysaccharides, carotenoids, and polyphenols, in modulating Aβ aggregation, mitigating tau protein pathology, and regulating gut–brain axis dysfunction. Furthermore, the challenges of current research are discussed, with an emphasis on improving blood–brain barrier permeability and optimizing drug delivery systems to facilitate clinical translation.

## 1. Introduction

Alzheimer’s disease (AD) is an irreversible neurodegenerative condition and the predominant form of dementia, representing a principal health challenge for the elderly population [1]. According to the 2024 *Alzheimer’s & Dementia* published data, approximately 6.9 million residents aged 65 and older in the United States have been diagnosed with AD, with projections indicating an increase to 13.8 million by 2060 if no new preventive, mitigating, and therapeutic interventions are implemented [2]. These statistics underscore the critical need for advancing research into novel therapeutic methodologies to address AD.

In 1907, Alois Alzheimer identified two key pathological features of the AD: extracellular amyloid plaques and intracellular neurofibrillary tangles (NFTs) [3]. Amyloid precursor protein (APP), a transmembrane glycoprotein, undergoes cleavage via two main pathways. The α-secretase pathway generates non-amyloidogenic fragments, while the β-secretase (BACE1) pathway produces sAPPβ and C-terminal fragment beta (CTFβ), which is further processed by γ-secretase to generate amyloid-beta (Aβ) peptides, including the neurotoxic Aβ42 that readily forms plaques [4,5,6]. Tau protein solubility is regulated by autophagic–lysosomal and ubiquitin–proteasome pathways, but dysfunction leads to abnormal phosphorylation and tangle formation [7]. Aβ and tau proteins synergistically drive AD progression, exacerbating its pathology [8].

Alzheimer’s disease pathology, while traditionally linked to limbic and cortical regions like the hippocampus, increasingly reveals central nervous system (CNS) alterations extending to the cerebellum. Although less studied than cerebral areas, cerebellar structural and functional changes correlate with cognitive decline, as evidenced by neuroimaging showing its dynamic biochemical involvement in AD progression [9]. Notably, Chen et al. [10] found that cerebellar models surpass hippocampal biomarkers in predicting early cognitive impairment, highlighting its diagnostic potential.

In patients with AD, the balance between Akt and GSK-3β activity is disrupted, with overactive GSK-3β promoting the formation of phosphorylated tau, increased Aβ production, neuronal dysfunction, and cell death [11]. Consequently, the inhibition of GSK-3β has emerged as a pivotal therapeutic target. Studies have demonstrated that specific inhibition of GSK-3β in APP23/PS45 bitransgenic mice reduces the transcription and expression of the BACE1 gene, thereby diminishing BACE1-mediated APP cleavage and Aβ generation [12]. Some of the marine natural products targeting GSK-3β-regulated AD will be mentioned below.

Additionally, AD is associated with neuroinflammation [13], oxidative stress [14], cellular senescence [15], mitochondrial dysfunction [16], and the loss of cholinergic signaling [17].

Existing therapeutic approaches [18,19] largely target singular aspects of AD pathology, such as Aβ aggregation, yet they have demonstrated limited clinical efficacy and often come with considerable side effects. This highlights the urgent need to explore novel, multi-targeted therapeutic strategies that address the multifaceted nature of AD pathology. However, Aβ and tau undoubtedly remain two important targets for the treatment of AD.

Marine natural products present remarkable therapeutic advantages, including broad availability, low toxicity, and environmental sustainability. As the largest global ecosystem, covering over 70% of the Earth’s surface and accounting for 90% of the planet’s biomass, the ocean serves as an unparalleled reservoir of biodiversity. Marine organisms, such as algae and sponges, have adapted to extreme environments, evolving unique metabolic pathways that make them exceptional sources for the discovery of innovative and effective drugs [20]. Researchers have uncovered a plethora of unique and diverse structures in the marine environment, including polysaccharides, carotenoids, polyphenols, sterols, and alkaloids, which exhibit distinct biological and pharmacological activities [21]. Indeed, certain studies have shown that natural products extracted from the ocean possess anti-inflammatory [22], antioxidant [23], antitumor [24], and antihypertensive properties [25]. Some marine natural products have already been approved for clinical use, such as Aplidin, a marine drug extracted from *Aplidium albicans*, used for the treatment of multiple myeloma [26]; Ziconotide, a non-opioid drug derived from the venom of the sea snail Conus magus, is employed for the treatment of severe chronic neuropathic pain [27]. In the context of AD therapy, marine-derived natural products have demonstrated potent effects [28,29,30], and as the first marine-derived drug approved for the treatment of AD, GV-971 is extremely important in linking marine ecosystems and Alzheimer’s disease treatment [31]. Its pharmacological effects will be detailed in Section 7 of this review.

Moreover, marine natural products hold significant potential for both scientific discovery and economic development. Currently, only GV-971, a marine-derived drug, has been marketed in China for AD, with others still in the animal testing phase. This highlights the vast untapped market potential. Further development of marine-based pharmaceuticals and nutraceuticals can drive economic growth, create jobs, and enhance global competitiveness in biotechnology and healthcare. Additionally, sustainable use of marine resources can support local economies and promote environmental sustainability.

This review examines the therapeutic potential of marine-derived compounds in Alzheimer’s disease, focusing on their ability to target key pathological processes. By integrating ADMET analysis, it highlights challenges such as blood–brain barrier (BBB) permeability and drug delivery, providing a foundation for developing novel, clinically translatable treatments.

To ensure the references are relevant and within the scope of this review, we searched PubMed using keywords such as “marine natural products”, “seaweed”, “sponge”, and “Alzheimer’s disease”. We also explored the references of key studies and identified additional works by notable authors. This approach allowed us to gather the literature both horizontally and vertically. Finally, we screened the studies to include only those published between 2019 and 2025, ensuring the review’s relevance and scientific rigor. Moreover, we specifically selected studies that focused on marine-derived compounds with demonstrated or potential therapeutic effects on Alzheimer’s disease, thereby ensuring that all included references are pertinent to our research focus.

Given the still-unclear mechanisms of AD and the multi-target effects of marine natural products, we will subsequently categorize these products based on the distinct pathological mechanisms of AD and their corresponding targets. This approach aims to provide a comprehensive understanding of the interactions between marine natural products and AD.

## 2. Marine Natural Product-Induced Regulation of Aβ

To understand the pathological mechanisms underlying AD and the potential therapeutic targets for intervention, it is essential to examine the role of Aβ peptides in the disease process. Within the cerebral matrix, APP is cleaved by specific enzymes, yielding Aβ peptides of varying lengths [32]. Under normal circumstances, these peptides remain soluble and are readily metabolized by cellular processes. In pathological states, however, Aβ peptides aggregate into soluble oligomers and fibers, which subsequently transition into insoluble amyloid plaques [33]. These aggregates, particularly in oligomeric form, exert potent neurotoxicity, disrupting normal neuronal functions, impairing synaptic transmission, and compromising cognitive abilities, such as learning and memory.

In fact, AD can be categorized into familial (FAD), which accounts for 1–5% of AD cases, and sporadic forms (SAD), which constitute over 95% of cases [34]. In SAD, apolipoprotein E (APOE), particularly the ε4 allele, is the most critical genetic risk factor. Carrying one or two APOE ε4 alleles increases the risk of AD by 2–3 and 12-fold, respectively [35], which may be an important factor in promoting the insolubility of Aβ [36]. Notably, APOE ε4 can impair the function of microglia, which are responsible for clearing Aβ plaques in brain. Specifically, APOE ε4 has been found to promote the accumulation of lipid droplets in microglia, leading to their dysfunction and reduced ability to clear Aβ [37].

Therefore, curtailing the formation of Aβ oligomers constitutes a significant therapeutic target in the treatment of AD. This includes strategies such as Aβ immunotherapy, inhibitors of Aβ aggregation, allosteric modulators of γ-secretase, and enzymes that degrade Aβ [38]. Encouraging clinical data from initial human trials in AD appear to endorse the potential of Aβ-targeted immunotherapy, which is capable of neutralizing these oligomeric species [39].

Natural products derived from marine organisms show promise in regulating Aβaggregation and mitigating its neurotoxicity. For example, molecular docking and dynamic simulation analyses have demonstrated that phlorofucofuroeckol-A (PFFA) and other phlorotannins, such as eckol, dioxinodehydroeckol, dieckol, and phloroglucinol, extracted from the brown alga *Ecklonia stolonifera*, exhibit strong interactions with Aβ25–35, effectively inhibiting its self-assembly and conformational changes [40]. Fucosterol pretreatment reduces APP mRNA expression and Aβ levels in activated SH-SY5Y cells, thereby decreasing amyloid protein production [41]. Additionally, dieckol has been shown to reduce Aβ generation in SweAPP N2a cells by activating the PI3K/Akt signaling pathway, subsequently inhibiting GSK-3β [42]. These findings suggest that targeting the upstream Aβ may be a key mechanism of action for marine natural products.

In Alzheimer’s patients, insulin resistance in the brain, particularly in the hippocampus, characterized by elevated insulin receptor substrate 1 (IRS-1) serine phosphorylation [43], is linked to disrupted insulin signaling and reduced PI3K/Akt activity. This imbalance results in GSK-3β activation and increased Aβ production [44]. Astaxanthin significantly reduces solubility levels in the hippocampus and dose dependently mitigates hippocampal IRS-1 phosphorylation, exerting neuroprotective effects [45].

In Alzheimer’s patients, the disruption of the balance between α-secretase and non-α-secretase pathways leads to increased BACE1 activity, resulting in Aβ accumulation [46]. Therefore, inhibiting BACE1 activity is a critical therapeutic target in the treatment of Alzheimer’s disease. Fucofuroeckol-b, isolated from *Eisenia bicyclis*, exhibits potent BACE1 inhibitory activity (IC50 of 16.1 μM) and functions through a non-competitive inhibitory mechanism, thereby reducing insoluble Aβ production [47]. Similarly, in the 5XFAD mouse model, phloroglucinol significantly reduced BACE1 levels and Aβ burden, improving cognitive function and synaptic plasticity. Notably, oxidative stress activates BACE1 through reactive oxygen species (ROS) accumulation, highlighting this process as a potential therapeutic target for AD intervention [48]. BACE2, a homolog of BACE1, plays an opposite role in Aβ generation despite their structural similarities. BACE1 cleaves APP at the β-site to produce Aβ peptides, including the aggregation-prone Aβ42. In contrast, BACE2 cleaves APP at the θ-site, producing a fragment (C80) that is not efficiently converted to Aβ, thus reducing Aβ production. However, under specific conditions, BACE2 can produce Aβ. This reflects their distinct enzymatic properties and substrate preferences [49]. Current BACE inhibitors targeting BACE1 risk side effects that reduce their efficacy. Future drug designs should focus on selectively differentiating BACE1 and BACE2 activities to improve therapeutic precision.

The remainder of this section covers marine nature products targeting Aβ biochemical pathways associated with AD pathology, as summarized in Table 1.

Marine natural products, as highlighted above, offer a promising avenue for AD treatment by targeting Aβ aggregation and its associated pathologies. Nevertheless, it is essential to recognize the limitations of these studies. Future research should aim to go beyond the Aβ-centric theory, focusing on upstream regulators of Aβ to identify novel strategies for improving AD outcomes and mitigating the adverse effects of Aβ. Additionally, this approach should be complemented by investigations into other potential mechanisms, such as tauopathy and neuroinflammation, to achieve a more comprehensive understanding of AD pathogenesis.

## 3. Marine Natural Products Regulate Tau Protein

Another critical target in the pathogenesis of AD is tau protein, which, like Aβ, plays a significant role in disease progression. Tau protein, a pivotal microtubule-associated protein within the central nervous system [64], plays a crucial role in maintaining the structural integrity of neurons and facilitating intracellular substance transport under normal physiological conditions [65]. However, in the pathogenic process of AD, tau protein undergoes excessive phosphorylation and abnormal aggregation, culminating in the formation of NFTs [66]. These tangles impair normal neuronal function. Consequently, interventions targeting tau protein have become integral components of potential therapeutic strategies for AD.

Given the importance of tau in AD pathology, multiple marine natural products mitigate their aberrant phosphorylation by modulating related signaling pathways. Numerous studies have already demonstrated that Omega-3 fatty acids have beneficial effects on a variety of diseases [67], particularly in the case of AD, where DHA and EPA play significant roles in neuroprotection [68,69]. Omega-3 blood biomarkers are closely associated with brain glucose uptake, particularly in individuals at high risk for AD. By improving brain metabolism, DHA activates the PI3K/Akt pathway, which inhibits GSK-3β, a key enzyme responsible for tau protein phosphorylation. This suggests that Omega-3 may indirectly mitigate tau pathology and reduce tau protein aggregation [70]. A mixture of phlorotannin and fucoidan (comprises phlorotannin and fucoidan in a 4:6 ratio) extracted from *Ecklonia cava* activates the Akt signaling pathway, which, in turn, phosphorylates GSK-3β, leading to its inactivation. This inactivation of GSK-3β significantly reduces the hyperphosphorylation of tau protein, thereby mitigating the formation of NFTs and preserving the structural integrity of microtubules [71]. Qian et al.’s research [72] highlighted the cognitive improvement effects of cerebrosides derived from *Sea cucumbers* in mitigating Aβ1–42-induced deficits. It demonstrated that cerebrosides could attenuate excessive tau protein phosphorylation by modulating the PI3K/Akt/GSK-3β signaling pathway, thereby exhibiting significant neuroprotective potential in the context of Alzheimer’s disease.

Neuronal cholesterol accumulation promotes the formation of pathological neurofibrillary tangles, which are composed of misfolded phosphorylated tau (p-tau) proteins [73,74,75], and also enhances the accumulation of p-tau by inhibiting its proteasomal degradation [73]. Studies suggest that activating Liver X receptors (LXRs) reduces abnormal tau protein aggregation in tauopathy mouse models [76]. Additionally, lipids extracted from *Himanthalia elongata* were shown to regulate cholesterol metabolism, decrease tau phosphorylation, and enhance cognitive function in APPswePS1ΔE9 mouse models [77]. These findings underscore the therapeutic potential of targeting cholesterol metabolism to address tau-related neurodegeneration in Alzheimer’s disease.

Tau proteinopathy may propagate between neurons [78]. During the progression of AD, pathological tau protein spreads from one neuron to adjacent neurons, triggering abnormal phosphorylation and the aggregation of tau within these cells. The study conducted by Jin et al. [79] explored the effects of fucoidans on tau protein transmission. The results demonstrated that fucoidans exhibit the ability to competitively bind to tau protein, thereby preventing its surface attachment and subsequent internalization, indicating that fucoidans hold significant potential as therapeutic agents for inhibiting the propagation of tau protein.

Although tau protein is an important therapeutic target for AD, only a few compounds have been identified that can effectively target tau protein. This may be due to the complex pathological mechanisms of tau protein, and the research on how these compounds interact with tau protein is not yet sufficient. Moreover, the screening and evaluation of marine bioactive compounds typically require complex biochemical and pharmacological studies, which may limit the discovery and development of marine bioactive compounds targeting tau protein. Therefore, while actively exploring the mechanisms by which marine organisms treat AD, it is also essential to delve deeper into the mechanisms of interaction between tau protein and AD.

**Figure 1:**
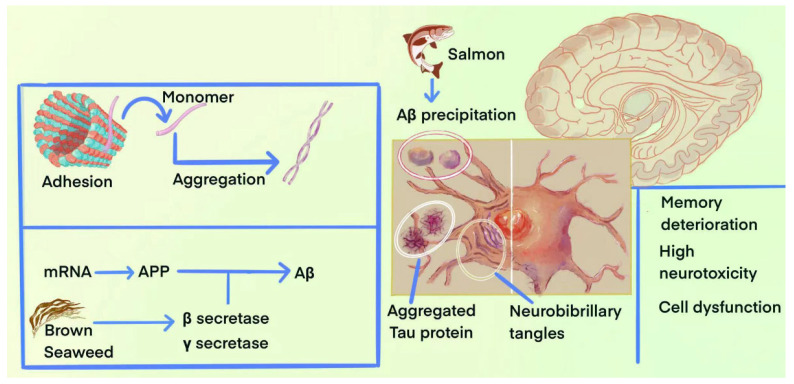
The mechanism of and marine natural products for regulating Aβ and tau.

The mechanism of and natural products for regulating Aβ and tau protein are shown as Figure 1. The remainder of this section covers marine natural products capable of improving AD by regulating tau protein phosphorylation, as summarized in Table 2.

## 4. Marine Natural Product-Induced Regulation of Cholinergic Functions

In the pathophysiology of AD, the impairment of the cholinergic system also plays a pivotal role [80]. In AD patients, the progressive degeneration of central cholinergic neurons results in the reduced activity of the enzyme choline acetyltransferase (ChAT), thus lowering the synthesis of acetylcholine (Ach) [81]. Concurrently, the increased activity of acetylcholinesterase (AChE) accelerates the breakdown of Ach [81], further exacerbating the deficiency. In some cases, butyrylcholinesterase (BChE), another major cholinesterase hydrolase enzyme, also acts equally; in healthy brains, AChE is the primary enzyme responsible for degrading ACh, while BChE plays a minor role. However, in AD, AChE activity declines by up to 45%, while BChE activity in the cortex and hippocampus can increase twofold. This shift makes BChE a more prominent target in the later stages of AD. Compared to AChE, BChE has broader substrate specificity [82,83], which means that inhibiting BChE could affect the metabolism of multiple cholinesterases and potentially lead to side effects. Therefore, when targeting BChE for inhibition, it is crucial to enhance its specificity for a single substance. This neurotransmitter deficiency undermines neuronal communication, disrupting the regulation of learning and memory. Consequently, enhancing ChAT activity while reducing AChE and BChE functions has become a critical strategy in AD treatment.

A mixture of phlorotannin and fucoidan extracted from *Ecklonia cava* exhibits anti-AD effects by inhibiting AChE and promoting ChAT expression, thereby protecting the brain’s cholinergic system [71]. The three main phlorotannins—eckol, dieckol, and 8,8′-bieckol—isolated from *Ecklonia cava* exhibit inhibitory effects on AChE. Among them, dieckol and 8,8′-bieckol demonstrate particularly strong activities against AChE while also showing notable inhibition of BACE1, with 11-hydroxy-8,8′-bieckol showing the strongest inhibition against BACE1 [54]. This dual-target inhibition suggests the potential for synergistic effects in addressing multiple aspects of AD pathology. Such a dual-action mechanism may offer superior efficacy compared to single-target therapies, highlighting the therapeutic promise of phlorotannins.

Fascaplysin, originally isolated from the Fijian sponge *Fascaplysinopsis* sp., has been shown to inhibit AChE activity and stimulate P-glycoprotein expression, providing neuroprotective effects [84]. In efforts to improve its cholinesterase inhibition, researchers have developed several derivatives, including 9-methylfascaplysin. Both Fascaplysin and 9-methylfascaplysin inhibit Aβ aggregation, with the latter showing increased potency [59]. Discorhabdin alkaloids extracted from the Antarctic *Latrunculia* spp. sponges demonstrate acetylcholinesterase inhibition [85]. The simplification of the compound based on its active moieties has led to the creation of derivatives with stronger AChE inhibition compared to Discorhabdin G, along with greater selectivity for AChE over BChE [86].

The structural features of nature compounds such as 11-hydroxy-8,8′-bieckol, 9-methylfascaplysin, and Discorhabdin G provide valuable insights into the relationship between their molecular architecture and inhibitory potency. These examples underscore the importance of structure–activity relationship analysis in drug design, particularly for AD therapies targeting key enzymes like AChE and BACE1.

Matrix metalloproteinases (MMPs) are a group of calcium-dependent zinc endopeptidases capable of degrading various components of the extracellular matrix, such as collagen, elastin, and fibronectin. Studies have shown that certain MMPs, including MMP-3, MMP-9, MMP-12, and MMP-13, contribute significantly to the progression of neurodegenerative diseases [87]. Interestingly, studies have demonstrated that treatment with astaxanthin, a natural antioxidant, effectively inhibits both MMP-13 and AChE activities [52].

**Figure 2:**
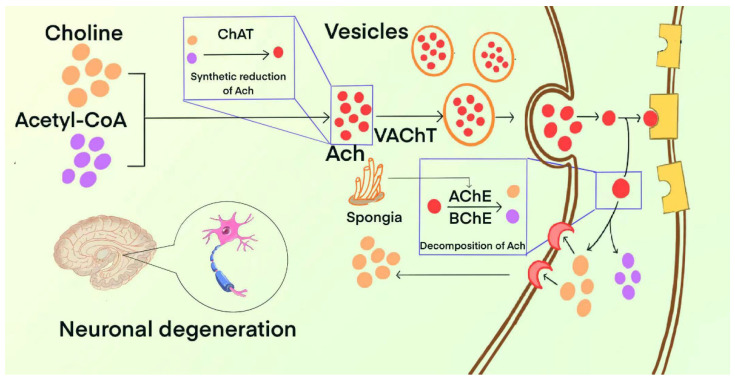
The mechanism of choline regulation by marine natural products.

The mechanisms of choline regulation by marine natural products are shown as Figure 2. The remainder of this section covers marine natural products capable of improving AD by regulating choline, as summarized in Table 3.

## 5. Marine Natural Products Inhibiting Neuroinflammation and Oxidative Stress

Neuroinflammation and oxidative stress have also been proven to play crucial roles in the development and progression of AD. In the early stages of AD, the accumulation of Aβ activates microglial cells, triggering the release of inflammatory cytokines such as TNF-α, IL-1β, and IL-6 [93]. This neuroinflammatory cascade exacerbates cognitive decline by impairing synaptic plasticity and neuronal function while promoting further Aβ production [94]. Chronic inflammation also contributes to tau protein hyperphosphorylation and aggregation, worsening neurodegenerative changes [94].

Oxidative stress plays a critical role in AD, interacting with neuroinflammation to form a vicious cycle. Aβ accumulation increases ROS, which damage cellular components, induce oxidative stress, and activate microglial cells, amplifying inflammation and ROS production [95]. The interplay between inflammation and oxidative stress leads to neuronal injury, synaptic loss, and progressive cognitive decline.

Marine natural products have shown significant potential in directly mitigating neuroinflammation and oxidative stress, as demonstrated in both in vitro and in vivo studies. Fucoxanthin exerts anti-neuroinflammatory effects by inhibiting the Akt/NF-κB and MAPKs/AP-1 pathways, thereby reducing the production of pro-inflammatory mediators, while simultaneously activating the Nrf-2/HO-1 and PKA/CREB pathways to enhance the expression of antioxidants and neurotrophic factors [96]. Similarly, ulvan reduces intracellular ROS levels and protects PC12 cells from oxidative stress-induced injury [56]. Mannan oligosaccharide (MOS), a polymer produced through the enzymatic depolymerization of alginate-derived poly-mannuronic acid (PM), diminishes the overexpression of TNF-α and IL-6 in the brains of AD mice, effectively mitigating both neuroinflammation and oxidative damage [97].

Transglutaminase 2 (TG2) is a calcium-dependent protein that is widely expressed in various tissues. It catalyzes cross-linking reactions that lead to the oligomerization and aggregation of Aβ [98]. This aggregation of Aβ not only exacerbates neuroinflammation but may also induce neuronal apoptosis. Astaxanthin pretreatment reduces the overexpression of TG2, GFAP, and vimentin, inhibits cyclin D1 levels, and inhibits the activation of apoptotic pathways, significantly decreases ROS levels induced by Aβ exposure, and enhances cell survival [99].

During the process of protein glycosylation, advanced glycation end products (AGEs) are generated and found to accumulate in the brains of elderly individuals and those with AD [100]. The AGE-β pathway has the potential to activate GSK-3β, triggering Aβ aggregation and the hyperphosphorylation of tau while inducing oxidative stress and neuroinflammation through NF-κB activation [101]. PFFA, extracted from the *Ecklonia stolonifera*, inhibits non-enzymatic glycation induced by d-ribose and d-glucose in a dose-dependent manner, thereby reducing the formation of AGEs [40]. Trigonelline, a pyridine-based alkaloid found in Anemonia sulcata, Arbacia lixula, and Velella velella [102], has been shown to inhibit the formation of AGEs in vitro, reduce AGE levels, and mitigate oxidative stress, thereby reversing cognitive impairment in a D-galactose(D-Gal)-induced model [103].

As previously mentioned, hyperactive GSK-3β contributes to the formation of phosphorylated tau, increased Aβ production, neuronal dysfunction, and cell death. Eckmaxol, extracted from the brown alga *Ecklonia maxima*, significantly reverses the expression of phosphorylated GSK-3β induced by Aβ oligomers, with a binding score of 5.09 in Surflex-Dock simulations, suggesting potential hydrogen bond formation with GSK-3β. This suggests that eckmaxol may exert its neuroprotective effects through direct binding to GSK-3β [104]. Similarly, meridianins, marine indole alkaloids isolated from the ascidian Aplidium [105], exhibit inhibitory activity against GSK-3β. This activity has been supported by docking calculations and molecular dynamics simulations, further emphasizing their therapeutic potential in AD [106].

GSK-3β plays a pivotal role in the development of AD, contributing to key pathological processes such as tau hyperphosphorylation, Aβ aggregation, neuroinflammation, and oxidative stress. Its involvement in these interconnected mechanisms makes it a crucial target for potential treatments. Efforts to develop GSK-3β inhibitors, along with approaches to regulate its activity through upstream pathways, offer promising avenues for addressing the complex neurodegenerative changes in AD.

The remainder of this section covers marine natural products capable of improving AD by regulating neuroinflammation and oxidative stress hypothesis, as summarized in Table 4.

## 6. Marine Natural Products Regulating Mitochondrial Function

Mitochondrial dysfunction, as a downstream event of Aβ accumulation, is equally indispensable in the pathogenesis of AD. The accumulation of amyloid β-protein is a hallmark of AD, with the capability to directly disrupt mitochondrial function. Aβ can insert into the mitochondrial membrane, leading to a loss of membrane potential, affecting ATP synthesis, and concurrently inducing the mitochondria to produce an excess of ROS [116]. Moreover, the abnormal phosphorylation and aggregation of tau protein can impair mitochondrial transport and distribution, leading to mitochondrial dysfunction, which further affects neuronal health [116].

Mitochondrial dysfunction is also implicated in neuroinflammation [117], where inflammatory responses exacerbate mitochondrial damage, creating a vicious cycle. Thus, maintaining mitochondrial health is of paramount importance for the prevention and treatment of AD, making strategies aimed at enhancing mitochondrial function a pivotal therapeutic approach.

Caspase-3 is a pivotal enzyme in the apoptosis process; upon receipt of apoptotic signals, increased mitochondrial membrane permeability results in the release of cytochrome c from the intermembrane space into the cytoplasm [118]. Bcl-2 and Bax, as anti-apoptotic and pro-apoptotic molecules, respectively, modulate mitochondrial permeability by forming pores or channels in the outer mitochondrial membrane, thereby influencing the apoptotic process. Studies have shown that a mixture of phlorotannin and fucoidan extracted from *Ecklonia cava* can enhance mitochondrial function by inhibiting the expression of molecules such as Bax, Cytc, and caspase-3 [71]. Treatment with fucoxanthin significantly promotes the recovery of mitochondrial function and regulates the balance between Bcl-2 and Bax [119]. Both DHA-PS and EPA-PS can markedly improve abnormal cellular morphology and facilitate the restoration of neural network structure, significantly reducing and inhibiting oxidative stress-mediated mitochondrial dysfunction [120]. However, DHA-PS exhibits a superior inhibitory effect on caspase-3 expression compared to EPA-PS, whereas EPA-PS shows a greater reduction in Bax/Bcl-2 levels than DHA-PS [120].

Astaxanthin exerts its therapeutic effects in AD primarily through the activation of the mTOR signaling pathway. This activation upregulates the expressions of mitochondrial fusion proteins, including Mfn1, Mfn2, and Opa1, while downregulating the expressions of mitochondrial fission proteins, such as Drp1 and Fis1, by modulating mitochondrial dynamics, enhancing synaptic plasticity, and reducing the accumulation of Aβ plaques [51]. Astaxanthin also modulates calcium influx via amalgamate receptors on the plasma membrane and improves mitochondrial function by reducing calcium uptake, thereby protecting neurons from excitotoxicity [121].

**Figure 3:**
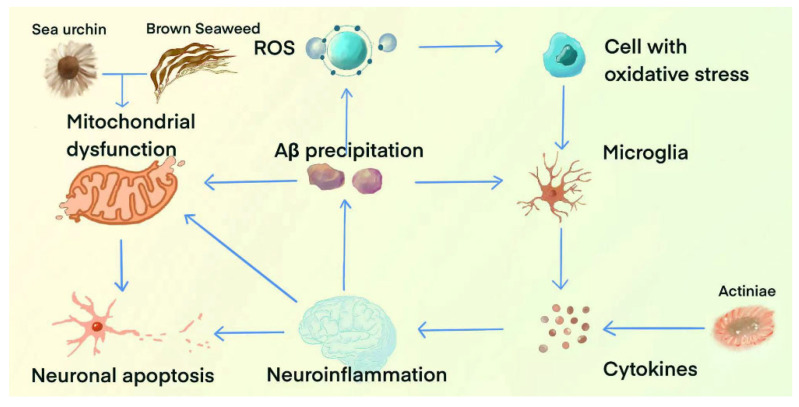
The mechanism of and marine natural products for regulating neuroinflammation, oxidative stress, and mitochondrial function.

In conclusion, marine natural products regulate mitochondrial function through several mechanisms, including the inhibition of the expression of apoptosis-related proteins, the activation of mTOR signaling, and the improvement of mitochondrial dynamic balance. The mechanism of and natural products for regulating neuroinflammation, oxidative stress, and mitochondrial function are shown as Figure 3. The remainder of this section covers marine natural products capable of improving AD by regulating mitochondrial dysfunction, as summarized in Table 5.

## 7. Marine Natural Products Regulating Gut Microbiota

In recent years, the gut microbiota has emerged as a focal point in AD research. Studies suggest that dysbiosis of the gut microbiota may be associated with central nervous system disorders such as AD [122], establishing a gut–brain axis through the production of metabolic products and interactions with the nervous, immune, and endocrine systems [122]. During this process, metabolic products of the gut microbiota can traverse the gut barrier, enter the bloodstream, and permeate the blood–brain barrier to directly modulate neuronal function while also influencing neuronal function by regulating the immune system [123]. Previous research has shown that bacterial metabolic products, particularly short-chain fatty acids (SCFAs) and bile acids, are directly or indirectly involved in certain neurological diseases [124,125]. Moreover, gut microbiota can affect the synthesis and release of neurotransmitters such as gamma-aminobutyric acid (GABA), 5-hydroxytryptamine (5-HT), and acetylcholine, which play crucial roles in regulating mood, cognitive functions, and sleep [126].

Studies have shown that MOS restructured the community of gut microbes, leading to an increase in the proportion of Lactobacillus species and a decrease in the proportion of Helicobacter species. Additionally, MOS boosted the production of butyrate and elevated the population of microbes associated with this process [97].

Based on the critical role of the gut microbiota in AD pathogenesis, GV-971 is a blend of acidic linear oligosaccharides, ranging from dimers to decamers (with molecular weights of up to ~1 kDa), derived from marine brown algae extracts [31]. GV-971, effectively restores gut microbiota and reduces the phenylalanine and isoleucine levels in both feces and blood. It also diminishes T-helper cell 1 (Th1)-related neuroinflammation and mitigates Aβ deposition as well as tau protein hyperphosphorylation [127]. However, animal studies have shown that GV-971 has sexual differences, exhibiting more pronounced effects in male mice compared to female mice [128].

Recently, a novel study has found that GV-971 binds directly to the GIANLDKL amino acid region of the Rib adhesin, blocking the adhesion of the Ribhigh-L.m strain to intestinal epithelial cells, correcting the accumulation of excessive lactate, inhibiting the GPR81-NFkB-mediated production of serum amyloid A (SAA), and ultimately alleviating the inflammatory response induced by Th1 cells in 5XFAD transgenic mice [129].

Clinical trials have validated the efficacy of GV-971. In a randomized, double-blind, placebo-controlled, multicenter phase III trial conducted in China (NCT 02293915), a total of 818 participants were randomized to receive either 900 mg of GV-971 (n = 408) or a placebo (n = 410). Importantly, the incidence of treatment-emergent adverse events was comparable between the active treatment and placebo groups, with rates of 73.9% and 75.4%, respectively. Notably, two deaths occurred in the GV-971 group, but these were determined to be unrelated to the drug effects. Furthermore, GV-971 demonstrated significant benefits in improving the cognitive function in patients with mild to moderate AD dementia. Specifically, this improvement was evident at the first assessment and persisted throughout the entire 36-week trial [130]. GV-971 has been approved for marketing by the China National Medical Products Administration [31] and included in the national healthcare insurance directory for the treatment of patients with mild to moderate AD [31], marking the first new drug targeting the brain–gut axis for AD both in China and globally; unfortunately, GV-971 was only approved in China.

Although GV-971 shows some efficacy in clinical trials, there are still some limitations that suggest further investigation. First, one limitation of the aforementioned clinical trial was the lack of a requirement for the presence of a diagnostic amyloid biomarker at screening, which could have potentially allowed participants with dementia caused by non-amyloid-related diseases to be included. Additionally, while the international phase III clinical study of GV-971 was approved by the regulatory agencies of 11 countries/regions and conducted in 162 centers worldwide as a multicenter, double-blind, placebo-controlled study, it was prematurely terminated for various reasons (FDA IND: 144482; NMPA Approval: CXHB2000033). Moreover, the mechanism by which GV-971 affects the development of AD through the gut microbiota is still under investigation, and the safety of GV-971 requires further validation through additional clinical trials.

Based on these challenges, GV-971 requires longer-term and larger-scale clinical trials to verify its long-term efficacy and safety. Trials should be conducted in diverse regions and populations to assess its generalizability. Additionally, it is crucial to elucidate the relationship between GV-971, the gut microbiota, and AD, particularly its impact on specific bacterial communities and how these communities influence neuroinflammation and cognitive function. These areas may represent the future direction of efforts for GV-971 and even for the development of new AD treatments.

The gut microbiota, a focal point of current research, has been closely linked to a multitude of diseases [131]. The gut–brain axis, as a novel target for AD treatment [132], offers a fresh direction for exploring the mechanisms by which marine organisms may treat AD. The success of GV-971 further clinically validates this target. In future research, it is recommended to strengthen the study of the interaction between gut microbiota and marine natural products, especially their joint impact on the pathological process of AD. This may involve using animal models and clinical trials to assess the regulatory effects of marine natural products on the composition and function of the gut microbiota, as well as how these changes influence the progression of AD and treatment response. Through such research, we can better understand the potential of marine natural products in AD treatment and provide a scientific basis for developing more effective therapeutic strategies.

## 8. ADMET Analysis

To fully harness the therapeutic potential of marine natural products for the treatment of AD, it is imperative to elucidate the relationship between their chemical structures and pharmacological properties. In this section, we will use phlorotannins as an example to illustrate how ADMETlab 3.0 can be employed to analyze and elucidate the correlation between their chemical structures and pharmacological profiles. Phlorotannins, a class of polyphenolic compounds derived from brown algae such as *Ecklonia bicyclis* and *Ecklonia cava*, are synthesized from phloroglucinol through C–C and/or C–O–C linkages [133] to form unique phenolic structures [134]. Based on their degree of polymerization and structural diversity, phlorotannins are categorized into six distinct groups: phloroethols, fuhalols, fucophloroethols, fucols, eckols, and carmalols [135]. This discussion focuses on two specific types: eckols and fucophloroethols.

**Figure 4:**
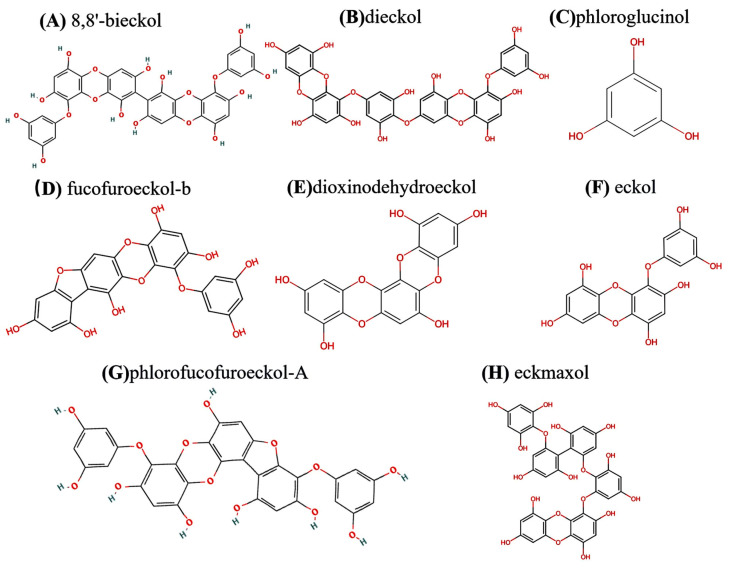
Structures of phlorotannins.

Eckols are distinguished by the incorporation of a 1,4-dibenzodioxin moiety within their molecular framework [136], as exemplified by compounds such as dieckol, dioxinodehydroeckol, eckol, phlorofucofuroeckol-A, and fucofuroeckol-b in Figure 4. Fucophloroethols, on the other hand, are characterized by the synthesis involving both ether and phenyl linkages, which can be observed in compounds such as fucophlorethol B, fucophlorethol A, and fucodiphlorethol B [136], including 8,8′-bieckol and eckmaxol in Figure 4.

To address AD, therapeutic agents must traverse the BBB to exert their effects within the central nervous system. As indicated in Table 6, although phlorotannins exhibit favorable lipid solubility, which generally facilitates BBB penetration, their capacity to cross the BBB remains suboptimal. This is likely due to other factors that can significantly influence their ability to reach the brain. This limitation arises from several key factors. Firstly, the large molecular weight and structural complexity of these compounds often lead to extensive hydrogen bonding, which can reduce lipid solubility and hinder their ability to cross the BBB. However, other factors, such as molecular rigidity and hydrophobicity, also play critical roles in determining BBB permeability. Secondly, their low P-glycoprotein (P-gp) inhibitory activity exacerbates this challenge. P-gp, which is highly expressed in the endothelial cells of the liver, kidney, intestine, and BBB, functions as an efflux transporter that reduces the intracellular concentrations of many drugs, potentially leading to therapeutic failure [137]. While effective AD therapies often involve compounds capable of modulating P-gp activity to sustain drug efficacy, phlorotannins display low P-gp inhibition and high P-gp substrate activity, further limiting their effectiveness in vivo. Finally, the fraction unbound (Fu) of these phenolic compounds is typically less than 40%, with a plasma protein binding (PPB) rate of approximately 90%. This high binding affinity means that only a small proportion of these compounds exist in their free form, which is necessary for therapeutic activity. Additionally, the high PPB may influence their pharmacokinetic properties, such as their half-life or elimination rate, and could also contribute to their reduced permeability across the BBB. Consequently, this may affect their central nervous system efficacy. In conclusion, while phlorotannins demonstrate promising therapeutic properties, overcoming these limitations—through strategies such as molecular modification, advanced drug delivery systems, or the modulation of P-gp activity—will be essential for improving their bioavailability and BBB permeability, thereby enhancing their potential as AD treatments.

Enhancing the blood–brain barrier permeability of marine-derived compounds is crucial for improving their therapeutic potential in Alzheimer’s disease treatment. Despite their unique advantages, such as lower systemic toxicity, these compounds face challenges including poor bioavailability and limited CNS penetration. Future research should prioritize optimizing these properties through advanced drug delivery systems, chemical modifications, and rigorous validation in animal or cell-based experiments, paving the way for clinical translation. The ADMET properties and structures of the other marine nature products mentioned in the review are visible in the Supplementary Materials.

## 9. Conclusions and Perspectives

Natural bioactive compounds derived from marine ecosystems, such as polysaccharides, polyphenols, sterols, carotenoids, diterpenes, and alkaloids, have garnered significant attention for their remarkable neuroprotective properties and low toxicity. These compounds, isolated from diverse marine organisms, hold immense potential for the development of novel therapeutic agents, particularly in the context of neurodegenerative diseases like AD. Their multifaceted biological activities suggest that they could serve as effective interventions to mitigate neuronal damage and promote cognitive health.

Nevertheless, the complete realization of marine bioresources’ therapeutic potential is still impeded by several unresolved challenges, among which extraction efficiency and blood–brain barrier permeability are particularly significant.

Initially, due to the complex mixture of bioactive compounds present in marine organisms, the yield of isolated bioactive substances is often low, precluding accurate assessments of their biological activity. For instance, substantial amounts of fucosylated sulfated polysaccharides and alginates are embedded within cell walls, cross-linked with cellulose, and phlorotannins form covalent and non-covalent bonds with proteins, presenting significant obstacles to the extraction of polyphenolic compounds [139]. Enhanced extraction technologies, such as high-pressure homogenization and supercritical fluid extraction, could improve yields and maintain bioactivity, enabling more precise evaluations.

Moreover, like other natural products, for marine drugs to exert their therapeutic effects on the nervous system, they must possess the ability to traverse the BBB and address issues related to P-gp-mediated drug efflux. Polyphenolic compounds have been shown to exert neuroprotective effects through the BBB into the central nervous system [140]. A recent study has demonstrated an innovative approach using genetically modified Toxoplasma gondii to cross the BBB and successfully deliver therapeutic proteins to neural cells in the brain, exhibiting superior delivery efficiency and functional recovery in both in vitro and in vivo models without significant toxicity [141], providing a new research direction for developing novel drug delivery systems. And emerging miniaturized therapeutic systems integrated with ultrasound technology present a novel platform for sustained and precise delivery of marine-derived compounds [142]. These systems hold the potential to enhance drug stability and ensure consistent therapeutic effects by targeting specific brain regions over extended periods

Ultimately, the development of marine-derived pharmaceuticals demands a multifaceted approach that integrates medical and pharmaceutical expertise with a deep understanding of marine science. This interdisciplinary requirement places substantial demands on researchers and significantly escalates the intricacy of marine drug development. The successful development of GV-971, a marine oligosaccharide for Alzheimer’s disease, serves as a powerful testament to the vast potential of marine compounds. It also underscores the critical need for collaborative efforts among marine scientists, biotechnologists, and clinicians.

To surmount these challenges, it is imperative to enhance interdisciplinary collaboration by integrating cutting-edge technologies and sustainable practices. Such an approach not only accelerates the discovery and optimization of bioactive compounds but also ensures the responsible utilization of marine resources. Initiatives such as establishing joint research platforms or utilizing artificial intelligence for the screening of bioactive compounds offer promising pathways to bridge the gap between discovery and clinical application. By fostering these collaborative and innovative strategies, we can unlock the therapeutic potential of marine bioresources and pave the way for groundbreaking advancements in medicine.

For example, this review utilizes ADMETlab 3.0 [143] to evaluate the pharmacological properties of marine natural products, enabling precise predictions of ADMET profiles such as BBB permeability, plasma protein binding, and toxicity. By leveraging advanced algorithms, ADMETlab effectively addresses the challenges associated with the structural complexity of marine compounds, facilitating the identification of promising drug candidates with optimized pharmacokinetics for clinical development.

The successful launch of GV-971, a pioneering marine drug for AD, is encouraging. Marine drugs show significant potential not only for treating neurodegenerative disorders but also for addressing various other diseases. Integrating marine bioresources into AD research, combined with advancements in extraction technologies and drug delivery systems, could unlock their full potential and drive major breakthroughs in marine pharmacology, offering innovative solutions for AD treatment. However, as previously mentioned, the clinical translation of marine natural products faces numerous challenges. Despite these obstacles, the potential of marine-derived compounds for AD treatment remains promising.

The exploration and utilization of marine resources offer substantial opportunities for scientific progress and economic expansion. However, it is crucial to weigh these activities against their environmental implications. As our understanding of marine biology and pharmacology deepens, the sustainable stewardship of these resources emerges as a pivotal concern. Looking ahead, it is imperative to embed sustainable methodologies within the procurement and application of marine-derived materials to curb ecological damage. This encompasses the implementation of cutting-edge technologies designed to reduce habitat interference, advocacy for regulations that curb excessive resource use, and the promotion of global collaboration to safeguard the enduring health of our oceans.

## Figures and Tables

**Table 1:** Marine natural product-induced regulation of Aβ.

Category	Compound	Origin	Test Model	Pharmacological Mechanism	References
Carotenoid	Astaxanthin	*Phaffia rhodozyma*	In vivo: female Albino rats were intracerebroventricularly administered with Aβ (1–42) dissolved in aCSF at a concentration of 4 μg/4 μL	Reduces IRS-1 phosphorylation, inhibits GSK-3β, and decreases Aβ aggregation	[45]
		-	In vivo: type 2 diabetes-induced APPxhQC transgenic and NTG mice, daily injections of 40 mg/kg streptozocin for 3 days	Levels of both soluble and insoluble Aβ are decreased	[50]
		-	In vivo: APP/PS1 transgenic mice, administered mTOR inhibitor rapamycin (3 mg/kg body weight, intraperitoneal injection) every third day	Enhances Aβ-degrading enzymes NEP and IDE expression	[51]
		-	In vivo: single injection of STZ (350 mg/kg, i.p.; and 2.5 μL, i.c.) in a zebrafish model	Reduces soluble Aβ levels	[52]
Polyphenol	Phlorofucofuroeckol-A, eckol, dioxinodehydroeckol, dieckol, and phloroglucinol	*Ecklonia stolonifera*	In vitro: the inhibition experiment of Aβ25–35 self-aggregation and non-enzymatic insulin glycation experiment	Prevents Aβ self-assembly and conformational changes	[40]
	Extraction of Symphyocladia latiuscula algae *	*Symphyocladia latiuscula*	In vitro: BACE1 enzyme inhibition assay	Inhibition of BACE1	[53]
	Eckol, diecko1, and 8,8′-bieckol	*Ecklonia cava*	In vitro: enzyme inhibition studies	Inhibition of BACE1 (8,8′-bieckol demonstrated the best inhibitory effect against BACE1 and AChE, with IC50 values of 1.62 ± 0.14 and 4.59 ± 0.32 μM, respectively)	[54]
	Eckol	*Ecklonia radiata*	In vitro: PC-12 cells with Aβ1–42 (0.05–1 μM)	Inhibition of Aβ amyloid toxicity	[55]
	Fucofuroeckol-b	*Ecklonia bicyclis*	In vitro: SH-SY5Y cells, exposed to Aβ42 for 24 h	Inhibition of BACE1 (IC50 of 16.1 μM)	[47]
	Phloroglucinol	*Ecklonia cava*	In vivo: 5XFAD mice	Inhibition of BACE1	[48]
	Dieckol	*Ecklonia cava*	In vitro: SweAPP N2a cells and N2a cells, maintained in 55% optiminimal essential medium and 45% Dulbecco’s modified Eagle’s medium with 10% fetal bovine serum and 1% penicillin/streptomycin in 5% CO_2_ at 37 °C	Activation of the PI3K/Akt signaling pathway, thereby inhibiting GSK-3β	[42]
Polysaccharide	Ulvan *	Green algae of the genus *Ulva*	In vitro: PC 12 cells, with Aβ (10 μL/well)	Inhibition of Aβ fibrogenesis and formation of Aβ oligomers	[56]
	Fucoidan	*Holothuroidea*	In vitro: PC-12 cells	Reduction in Aβ neurotoxicity by decreasing its inhibition of PKC phosphorylation	[57,58]
Alkaloid	9-methylfascaplysin	*Fascaplysinopsis* sp.	In vitro: SH-SY5Y cells, with Aβ oligomer	Inhibition of Aβ aggregation	[59]
	Fascaplysin	*Fascaplysinopsis* sp.	In vitro: SH-SY5Y cells, with Aβ oligomer	Inhibition of Aβ aggregation	[59]
	Aplysinopsins and its derivatives *	-	In vitro: BACE1 inhibition assay	Inhibition of BACE1 (IC50: 33.7 µM)	[60]
Sterol	24(S)-saringosterol	*Sargassum pallidum*	In vivo: male APPswePS1ΔE9 and wild-type (WT) littermates	Reduction in Aβ1–42 induced	[61]
	Fucosterol	*Sargassum pallidum*	In vitro: enzyme inhibition assay	Non-competitive inhibition of BACE1 activity	[62]
		*Fucus vesiculosus*	In vivo: sAβ1–42-induced cognitive impairment in aging rats	Inhibition of ERK1/2 signaling reduces cytotoxicity and suppresses GRP78 expression in hippocampal neurons	[63]
		*Fucus vesiculosus*	In vitro: in amyloid β-induced SH-SY5Y cells	Reduction in APP mRNA expression and Aβ levels in activated SH-SY5Y cells	[41]

* The substance is a mixture of many substances or a class of compounds.

**Table 2:** Marine natural products that regulate tau protein.

Category	Compound	Source	Test Model	Mechanisms	References
Polyphenol	P4F6 *	*Ecklonia cava*	In vivo: ICR mice (male, 4 weeks old), with Aβ1–42 injected	Activation of the Akt signaling pathway mitigates excessive phosphorylation of tau protein	[71]
Polysaccharide	Fucoidan	*Phaeophyceae*	In vitro: wild-type mouse lung endothelial cell lines, incubated with tau-AF488 (25 μg/mL) or tau-AF488 (25 μg/mL) mixed with heparin, SJ-D-I-H, SJ-GX-3, SJ-I, or SJ-D-I (25 μg/mL) at 37 °C and 5% CO_2_ for 3 h.	Competing with heparan sulfate for tau binding, thereby halting the dissemination of tau protein	[79]
Lipid	Lipid extract of *H. elongate* *	*Himanthalia elongata*	In vitro: cell lines HEK293, CCF-STTG1, SH-SY5Y, and CHME3 In vivo: APPswePS1ΔE9 mouse model	Reduction in hyperphosphorylation of tau protein	[77]
	Cerebroside	*Sea cucumber*	In vivo: male SD rats receive a ventricle injection of Aβ1–42 peptide	Activation of the PI3K/Akt/GSK-3β signaling pathway reduces tau hyperphosphorylation	[72]
	DHA	*Fatty fish*	In vivo: blood biomarkers and brain imaging data based on clinical data	Activation of the PI3K/Akt pathway suppresses GSK-3β activity, thereby reducing abnormal phosphorylation of tau protein	[70]

* The substance is a mixture of many substances or a class of compounds.

**Table 3:** Marine natural product-induced regulation of cholinergic functions.

Category	Compound	Origin	Test Model	Pharmacological Mechanism	References
Polyphenol	P4F6 *	*Ecklonia cava*	In vivo: ICR mice (male, 4 weeks old), with Aβ1–42 injected	Inhibition of AChE and enhancement of ChAT	[71]
	Extracts rich in phlorotannins (PEEC) *	*Ecklonia cava*	In vitro: neuronal PC-12 and SH-SY5Y cells, with 100 μM H_2_O_2_, AChE and BChE inhibition assays	Inhibition of AChE and BChE (IC50: AChE 68.9 μg/mL, BChE217.7 μg/mL)	[88]
	Extracts rich in phlorotannins (PFRI) *	*Ishige foliacea*	In vivo: male ICR mice, scopolamine i.p.	Inhibition of AChE activity in the brain	[89]
	Eckol, dieckol, and 8,8′-bieckol	*Ecklonia cava*	In vitro: enzyme inhibition studies	Inhibition of AChE (IC50: eckol, 5.28 μM; dieckol, 2.37 μM; 8,8′-bieckol, 1.45 μM)	[54]
	Bromophenols *	*-*	In vitro: enzyme inhibition assays	Inhibition of AChE	[90]
	Extract of S. latiuscula and three isolated 2,3,6-tribromo-4,5-dihydroxybenzyl derivatives (1, 2, and 3) *	*Symphyocladia latiuscula*	In vitro: cholinesterase enzyme inhibition assay	Inhibition of AChE (IC50: ***3***, 2.66 ± 0.24 μM; ***1***, 7.31 ± 0.25 μM; ***2***, 9.61 ± 0.35 μM) and BChE (IC50: ***3***, 4.03 ± 0.15 μM; ***1***, 8.95 ± 2.18μM; ***2***, 14.41 ± 0.27 μM)	[53]
Alkaloid	Fascaplysin and its derivatives *	*Fascaplysinopsis* sp.	In vitro: LS-180 cells	Inhibition of AChE (IC50 > 10 μM)	[84]
	Discorhabdin and its derivatives *	*Latrunculia biformis*	In vitro: cholinesterase inhibition	Inhibition of AChE and BChE, exhibiting a higher selectivity for AChE compared to BChE	[85,86]
	Trigonelline	-	In vivo: adult male Swiss albino mice, LPSs were dissolved in saline for oral (po) and intraperitoneal (i.p.) administration, respectively	Inhibition of AChE	[91]
	Petrosamine	*Petrosia* sp.	In vitro: SH-SY5Y cells, exposed to different concentrations of AlCl_3_ (50, 200, and 1000 μM) for 24 h; in vivo: zebrafish, induced by AlCl_3_	Strong inhibition of AChE (IC50: 0.091 μM)	[92]
	Aplysinopsins and its derivatives *	-	In vitro: AChE and BChE inhibition assays	Inhibition of cholinesterase; plysinopsin derivative 5b is a dual inhibitor of AChE and BChE (IC50 values of 3.9 and 14 μM)	[60]
Sterol	Fucosterol	*Fucus vesiculosus*	In vitro: enzyme inhibition assay	Inhibits BChE activity; serves as a non-competitive inhibitor for AChE	[62]

* The substance is a mixture of many substances or a class of compounds.

**Table 4:** Marine natural products that inhibit neuroinflammation and oxidative stress.

Category	Compound	Origin	Test Model	Pharmacological Mechanism	References
Polyphenol	P4F6 *	*Ecklonia cava*	In vivo: ICR mice (male, 4 weeks old), with Aβ1–42 injection	Activation of SOD and reduction in TBARS to mitigate oxidative stress	[71]
	PEEC *	*Ecklonia cava*	In vitro: neuronal PC-12 and SH-SY5Y cells, with 100 μM H_2_O_2_, AChE and BChE inhibition assays	Mitigation of oxidative stress	[88]
	PFRI *	*Ishige foliacea*	In vivo: male ICR mice, scopolamine i.p.	Reduction in lipid peroxidation levels, leading to increased GSH levels and enhanced SOD activity	[89]
	Phloroglucinol	*Ecklonia cava*	In vitro: primary cultured astrocytes, cells were treated with various concentrations (0.5–2 mM) of oAβ1–42 for 24 h	Reduction in GFAP protein expression levels, alleviating astrocyte activation; reduction in ROS to relieve oxidative stress	[107]
	Eckmaxol	*Ecklonia maxima*	In vitro: SH-SY5Y cells with Aβ oligomer	Binding with GSK-3β and inhibition of pGSK-3β production, thereby reducing intracellular ROS accumulation	[104]
	Phlorofucofuroeckol-A	*Ecklonia stolonifera*	In vitro: the inhibition experiment of Aβ25–35 self-aggregation and non-enzymatic insulin glycation experiment	Reduction in AGE formation, alleviating oxidative stress and neuroinflammation	[40]
	Eckol, dieckol, and 8,8′-bieckol	*Ecklonia cava*	In vitro: H_2_O_2_-stimulated cell damage in HT22 cells and PC12 cells, treated with Aβ25–35	Inhibition of iNOS and COX-2 expression, resulting in decreased protein-level production of TNF-α, IL-1β, and PGE2	[108]
Carotenoid	2% fucoxanthin PT extract	*Phaeodactylum tricornutum*	In vivo: male Swiss mice, subcutaneously injected with D-Gal at a dose of 150 mg/kg once per day, 5 days per week	Activation of Nrf2 to mitigate oxidative stress	[109]
	Fucoxanthin	*-*	In vitro: BV-2 cells, stimulated by LPS (100 ng/mL)	Activation of the Akt/GSK-3β/Fyn signaling pathway to reduce intracellular ROS levels; dose-dependent inhibition of inflammatory mediator secretion	[96]
	Astaxanthin	*Haematococcus pluvialis*	In vitro: ensheathing cells (OECs) were obtained from 2-day-old mouse	Reduction in TG2, GFAP, and cytoskeletal protein overexpression, inhibition of cyclin D1 levels, and activation of apoptotic pathways	[99]
		*Haematococcus pluvialis*	In vitro: PM2.5-induced BV-2 microglial cells	Inhibition of NF-κB and activation of Nrf2 nuclear translocation, reducing the expression of inflammatory mediators, thereby alleviating microglial activation	[110]
		-	In vitro: primary porcine brain capillary endothelial cells and murine organotypic hippocampal slice cultures	Anti-inflammatory effects by reducing the secretion of inflammatory cytokines and promotion of the polarization of microglia from M1 to M2	[111]
Alkaloid	Trigonelline	-	In vitro: bovine serum albumin In vivo: mice were subcutaneously (sc) injected with d-gal (150 mg/kg) for 6 weeks	Reduction in AGE levels, inhibition of oxidative stress, and exertion of neuroprotective effects	[103]
	Circumdatin D	*Aspergillus ochraceus LZDX32-15*	In vitro: BV-2 cells, induced by LPS In vivo: transgenic C. elegans CL4176 and E. coli OP50	Inhibition of TLR4-mediated NF-κB, MAPKs, and JAK/STAT inflammatory signaling pathways	[112]
	Meridianin A	*Aplidium savigny*	In vitro: cortical neuronal from E17.5 WT mouse embryos	Inhibition of GSK-3β activity, reducing microglial activation and astrocyte proliferation	[106]
	Lignarenone B	*Scaphanderlignarius*	In vitro: cortical neuronal from E17.5 WT mouse embryos	Inhibition of GSK-3β activity, reducing microglial activation and astrocyte proliferation	[106]
Polysaccharide	Ulvan	Green algae of the genus *Ulva*	In vitro: PC 12 cells, with Aβ (10 μL/well)	Downregulation of ROS production	[56]
	Mannan oligosaccharide *	*Alginic acid sodium salt*	In vivo: in the 5xFAD Alzheimer’s disease mouse model, MOS (0.12%, *w*/*v* in the drinking water)	Reduction in the overexpression of TNF-α and IL-6 in the brains of AD mice, alleviating neuroinflammation and oxidative damage	[97]
Amino acid	Taurine	-	In vivo: male Wistar rats received STZ (ICV, 3 mg/kg, bilateral, 5 uL per site, aCFS vehicle)	Upregulation of insulin receptor in the hippocampus and reduction in astrocyte proliferation in SDAT	[113]
		-	In vivo: senescence-accelerated mouse prone 8 (SAMP8) mice	Upregulation of TREM2 reduces the accumulation of Aβ and tau, which collectively alleviate the activation of microglia	[114]
	γ-GC	-	In vitro: Aβ oligomer (AβO) induced in microglia	Upregulation of Nurr1 protein expression, inhibiting NF-κB transcriptional activity on inflammatory genes	[115]
Lipid	Lipid extract of H. elongate *	*Himanthalia elongata*	In vitro: cell lines HEK293, CCF-STTG1, SH-SY5Y, and CHME3 In vivo: in the APPswePS1ΔE9 mouse model	Reduction in pro-inflammatory factor production in LPS-stimulated, THP-1-derived macrophages	[77]

* The substance is a mixture of many substances or a class of compounds.

**Table 5:** Marine natural products regulating mitochondrial function.

Category	Compound	Origin	Test Model	Pharmacological Mechanism	References
Polyphenol	P4F6 *	*Ecklonia cava*	In vivo: ICR mice (male, 4 weeks old), with injected Aβ1–42	Inhibit the expression of molecules such as Bax, Cytc, and caspase-3	[71]
Carotenoid	Fucoxanthin	-	In vitro: PC12 cells, with Aβ25–35	Increase Bcl-2 expression and reduce Bax expression	[119]
	Astaxanthin	-	In vivo: APP/PS1 transgenic mice	Activate mTOR, increasing the expression of mitochondrial fusion proteins and reducing the levels of mitochondrial fission proteins	[51]
		-	In vitro: cortical neurons obtained from postnatal day-one Wistar rat pups	Improve mitochondrial function by regulating calcium influx through glutamate receptors on the plasma membrane	[121]
Lipids	DHA and EHA	*Cucumaria frondose*; *Sthenoteuthis oualaniensis*	In vitro: hippocampal neurons were isolated from the brains of neonatal SD rats	Inhibit caspase-3 expression and reduce Bax/Bcl-2 levels	[120]

* The substance is a mixture of many substances or a class of compounds.

**Table 6:** ADMET analysis.

Parameters	8,8′-Bieckol	Dieckol	Phloroglucinol	Fucofuroeckol-b	Dioxinodehydroeckol	Eckol	Phlorofucofuroeckol-A	Eckmaxol
MW	742.08	742.08	126.03	478.05	370.03	372.05	602.07	744.1
LogS	−4.581	−4.194	−1.413	−3.704	−2.319	−3.26	−4.239	−3.966
LogP	2.626	2.177	0.329	2.329	1.231	1.351	2.335	1.241
Pgp-inh	−−−	−−−	−−−	−−−	−−−	−−−	−−−	−−−
Pgp-sub	−−	−	−−−	++	+	−	+	++
HIA	−−−	−−−	−−−	−−−	−−−	−−−	−−−	−−−
F (30%)	+++	+++	−	+++	+++	++	+++	+++
Caco-2	−7.659	−7.278	−5.065	−6.459	−5.707	−6.095	−6.692	−7.701
BBB	−−−	−−−	−−	−−−	−−−	−−−	−−−	−−−
PPB	95.4%	89.3%	52.8%	95.2%	93.4%	90.6%	93.1%	90.8
Fu	6.9%	13.1%	35.2%	7.0%	9.5%	12.8%	9.2%	12.2%
CYP1A2-inh	+++	+++	+++	+++	+++	+++	+++	+++
CYP1A2-sub	+++	+++	+++	+++	+	+++	+++	+++
CL	2.668	2.568	11.771	2.62	3.626	5.115	2.587	2.314
T_1/2_	4.165	3.778	1.332	2.598	1.744	1.959	3.402	4.02
hERG	0.178	0.291	0.146	0.168	0.047	0.187	0.208	0.241
Ames	0.508	0.683	0.477	0.736	0.765	0.639	0.712	0.701
ROA	0.573	0.534	0.464	0.677	0.633	0.468	0.585	0.604
FDAMDD	0.987	0.98	0.734	0.967	0.77	0.881	0.984	0.996
BCF	0.982	1.115	0.483	1.175	1.308	1.297	1.139	0.979

MW: molecular weight. LogS: the logarithm of the aqueous solubility value. LogP: the logarithm of the n-octanol/water distribution coefficient. Pgp-inh: the inhibitor of P-glycoprotein. Pgp-sub: the substrates of P-glycoprotein. HIA: human intestinal absorption. F (30%): the human oral bioavailability 30%. Caco-2: the permeability of human colon adenocarcinoma cell lines (Caco-2). BBB: the penetration of blood–brain barrier (BBB). PPB: plasma protein binding. Fu: the fraction unbound in plasma. CL: the clearance of a drug. T1/2: the half-life of a drug. hERG: the human ether-a-go-go-related gene. Ames: the Ames test for mutagenicity. ROA: the toxicity of rat oral acute. FDAMDD: the maximum recommended daily dose. BCF: the bioconcentration factor. For the classification endpoints, the prediction probability values are transformed into six symbols: 0–0.1 (−−−), 0.1–0.3 (−−), 0.3–0.5 (−), 0.5–0.7 (+), 0.7–0.9 (++), and 0.9–1.0 (+++). The data acquired from the ADMETLab 3.0 database. The table template comes from [138].

## Data Availability

Not applicable.

## Supplementary Materials

The following supporting information can be downloaded at: https://www.mdpi.com/article/10.3390/md23030091/s1, Figure S1: Structures of Carotenoid, Polysaccharide and Amino acid; Figure S2: Structures of Alkaloid; Table S1: ADMET analysis about Carotenoid, Polysaccharide and Amino acid; Table S2: ADMET analysis about Alkaloid.
