# Supplementary material for: The Advancements of Marine Natural Products in the Treatment of Alzheimer’s Disease: A Study Based on Cell and Animal Experiments
Source: Mar Drugs. 2025 Feb 20;23(3):91. doi: 10.3390/md23030091 (PMC11943648; doi:10.3390/md23030091)
Supplement: Supplementary file 1 [file marinedrugs-23-00091-s001.zip › marinedrugs-3425588-supplementary.pdf]

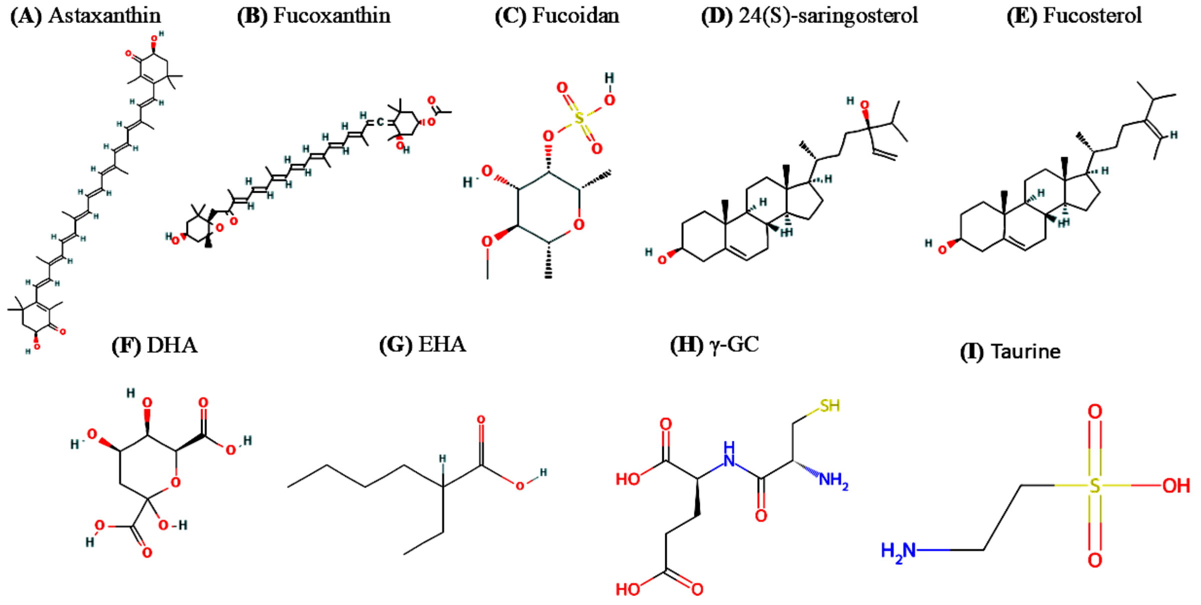

Supplementary Figure S1. Structures of Carotenoid,Polysaccharide and Amino acid.

Supplementary Table S1. ADMET analysis about Carotenoid,Polysaccharide and Amino acid.

Parameters	Astaxanthin	Fucoxanthin	Fucoidan	24(S)-saringosterol	Fucosterol	DHA	EHA	γ-GC	Taurine
MW	596.39	658.42	256.06	428.37	412.37	222.04	144.12	250.06	125.01
LogS	-5.212	-5.105	0.022	-6.288	-7.038	0.629	-1.428	-0.84	0.023
LogP	4.375	4.631	-0.21	6.891	7.696	-1.544	2.218	-1.313	-2.762
Pgp-inh	-	--	-	---	---	---	---	---	---
Pgp-sub	+++	+++	--	--	---	-	---	---	--
HIA	---	---	+++	---	---	---	--	--	+++
F(30%)	+++	+++	++	+	-	---	-	-	+++
Caco-2	-5.045	-4.95	-5.464	-5.209	-5.136	-6.226	-4.778	-6.175	-6.122
BBB	---	---	---	---	---	---	+	---	---
PPB	94.7%	91.0%	23.8%	81.4%	96.1%	21.6%	85.8%	15.9%	9.2%
Fu	5.5%	6.6%	82.4%	17.8%	5.1%	83.7%	13.2%	90.8%	89.5%
CYP1A2-inh	---	---	---	---	---	---	---	---	---
CYP1A2-sub	+++	---	---	---	---	---	---	---	---
CL	4.941	7.138	2.286	14.548	12.069	1.441	4.322	2.689	3.959
T1/2	0.885	0.779	1.812	0.093	0.545	3.444	1.125	1.969	1.882
hERG	0.046	0.048	0.032	0.137	0.129	0.003	0.048	0.000	0.046
Ames	0.89	0.706	0.911	0.166	0.086	0.214	0.077	0.514	0.285
ROA	0.833	0.627	0.25	0.167	0.094	0.051	0.148	0.128	0.111
FDAMDD	0.767	0.966	0.229	0.763	0.521	0.043	0.124	0.001	0.229
BCF	2.696	1.392	0.201	2.373	2.913	0.275	0.277	0.172	0.214

MW: molecular weight. LogS: the logarithm of the aqueous solubility value. LogP: the logarithm of the n-octanol/water distribution coefficient. Pgp-inh: the inhibitor of P-glycoprotein. Pgp-sub: the substrates of P-glycoprotein. HIA: human intestinal absorption. F (30%): the human oral bioavailability 30%. Caco-2: the permeability of human colon adenocarcinoma cell lines (Caco-2). BBB: the penetration of blood–brain barrier (BBB). PPB: plasma protein binding. Fu: the fraction unbound in plasma. CL: the clearance of a drug. T1/2: the half-life of a drug. hERG: the human ether-a-go-go-related gene. Ames: the Ames test for mutagenicity. ROA: the toxicity of rat oral acute. FDAMDD: the maximum recommended daily dose. BCF: the bioconcentration factor. For the classification endpoints, the prediction probability values are transformed into six symbols: 0–0.1 (−−−), 0.1–0.3 (−−), 0.3–0.5 (−), 0.5–0.7 (+), 0.7–0.9 (++), and 0.9–1.0 (+++). The data acquired from the ADMETLab 3.0 database.The table template comes from [138].

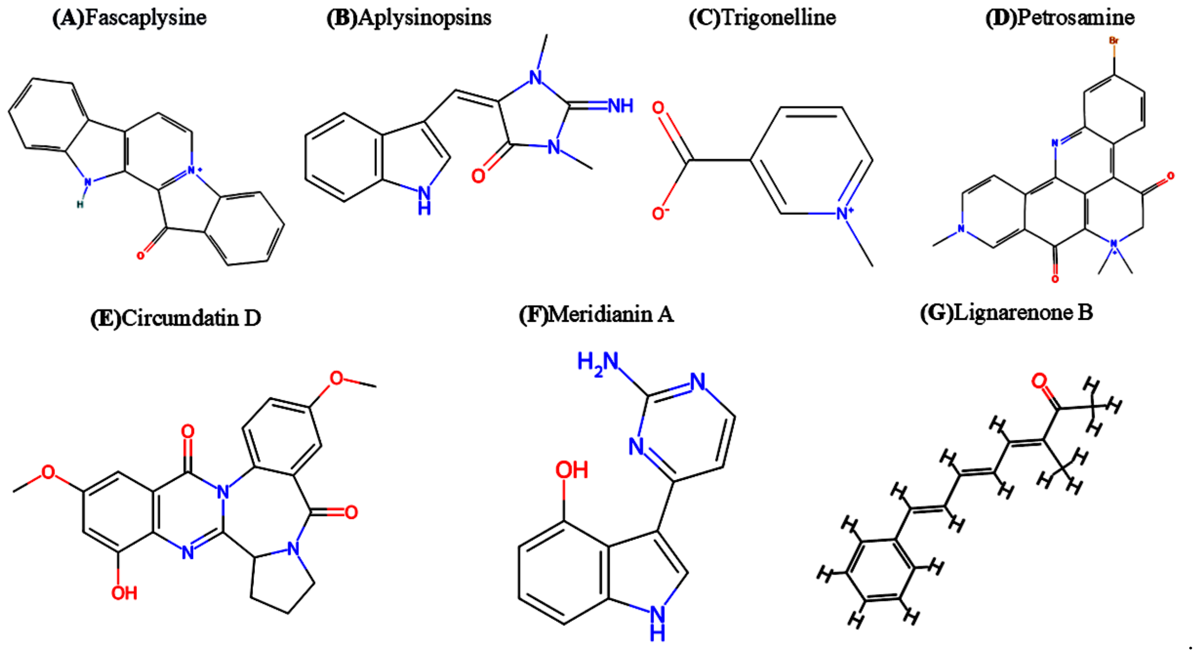

Supplementary Figure S2. Structures of Alkaloid.

Supplementary Table S2. ADMET analysis about Alkaloid.

Parameters	Fascaplysine	Aplysinopsins	Trigonelline	Petrosamine	Circumdatin D	Meridianin A	Lignarenone B
MW	271.09	254.12	137.05	137.05	393.13	226.09	212.12
LogS	-4.865	-2.701	-0.641	-0.641	-3.045	-2.772	-3.623
LogP	3.775	0.853	-0.612	-0.612	1.522	1.196	2.98
Pgp-inh	++	-	---	---	+++	---	+++
Pgp-sub	+++	---	---	---	+++	---	--
HIA	---	---	---	---	---	---	---
F(30%)	+++	--	++	++	-	++	-
Caco-2	-4.791	-5.126	-5.49	-5.49	-4.839	-5.132	-4.697
BBB	---	++	---	---	---	--	---
PPB	96.5%	90.5%	4.3%	4.3%	79.5%	67.9%	06.0%
Fu	3.3%	9.0%	91.7%	91.7%	16.3%	30.0%	4.7%
CYP1A2-inh	+++	+++	---	---	+++	++	+++
CYP1A2-sub	+++	+++	---	---	+++	---	--
CL	5.45	5.719	1.399	1.399	4.692	8.887	9.899
T1/2	1.457	0.94	3.219	3.219	0.902	0.943	0.941
hERG	0.443	0.545	0.08	0.08	0.365	0.093	0.183
Ames	0.915	0.83	0.181	0.181	0.855	0.831	0.788
ROA	0.867	0.619	0.289	0.289	0.439	0.667	0.448
FDAMDD	0.918	0.757	0.14	0.14	0.933	0.486	0.677
BCF	1.651	0.931	0.461	0.461	1.169	0.324	1.682

MW: molecular weight. LogS: the logarithm of the aqueous solubility value. LogP: the logarithm of the n-octanol/water distribution coefficient. Pgp-inh: the inhibitor of P-glycoprotein. Pgp-sub: the substrates of P-glycoprotein. HIA: human intestinal absorption. F (30%): the human oral bioavailability 30%. Caco-2: the permeability of human colon adenocarcinoma cell lines (Caco-2). BBB: the penetration of blood–brain barrier (BBB). PPB: plasma protein binding. Fu: the fraction unbound in plasma. CL: the clearance of a drug. T1/2: the half-life of a drug. hERG: the human ether-a-go-go-related gene. Ames: the Ames test for mutagenicity. ROA: the toxicity of rat oral acute. FDAMDD: the maximum recommended daily dose. BCF: the bioconcentration factor. For the classification endpoints, the prediction probability values are transformed into six symbols: 0–0.1 (−−−), 0.1–0.3 (−−), 0.3–0.5 (−), 0.5–0.7 (+), 0.7–0.9 (++), and 0.9–1.0 (+++). The data acquired from the ADMETLab 3.0 database.The table template comes from [138].
